# Prevalence of Clinical and Subclinical Myocarditis in Competitive Athletes With Recent SARS-CoV-2 Infection: Results From the Big Ten COVID-19 Cardiac Registry

**DOI:** 10.1001/jamacardio.2021.2065

**Published:** 2021-05-27

**Authors:** Curt J. Daniels, Saurabh Rajpal, Joel T. Greenshields, Geoffrey L. Rosenthal, Eugene H. Chung, Michael Terrin, Jean Jeudy, Scott E. Mattson, Ian H. Law, James Borchers, Richard Kovacs, Jeffrey Kovan, Sami F. Rifat, Jennifer Albrecht, Ana I. Bento, Lonnie Albers, David Bernhardt, Carly Day, Suzanne Hecht, Andrew Hipskind, Jeffrey Mjaanes, David Olson, Yvette L. Rooks, Emily C. Somers, Matthew S. Tong, Jeffrey Wisinski, Jason Womack, Carrie Esopenko, Christopher J. Kratochvil, Lawrence D. Rink

**Affiliations:** 1 Division of Cardiology, Department of Internal Medicine, Ohio State University, Columbus; 2 School of Public Health, Indiana University, Bloomington; 3 University of Maryland School of Medicine, Baltimore; 4 University of Michigan, Ann Arbor; 5 Indiana University School of Medicine, Bloomington; 6 University of Iowa Stead Family Children’s Hospital, Iowa City; 7 Ohio State University, Columbus; 8 Indiana University School of Medicine, Indianapolis; 9 Michigan State University, East Lansing; 10 University of Nebraska, Lincoln; 11 University of Wisconsin School of Medicine, Madison; 12 Purdue University, West Lafayette, Indiana; 13 University of Minnesota, Minneapolis; 14 Indiana University, Bloomington; 15 Feinberg School of Medicine, Northwestern University, Chicago, Illinois; 16 University of Maryland at College Park, College Park; 17 Penn State Health Sports Medicine, State College, Pennsylvania; 18 Robert Wood Johnson Medical School, Rutgers University, Newark, New Jersey; 19 Rutgers Biomedical and Health Sciences, Newark, New Jersey; 20 University of Nebraska Medical Center, Omaha

## Abstract

**Importance:**

Myocarditis is a leading cause of sudden death in competitive athletes. Myocardial inflammation is known to occur with SARS-CoV-2. Different screening approaches for detection of myocarditis have been reported. The Big Ten Conference requires comprehensive cardiac testing including cardiac magnetic resonance (CMR) imaging for all athletes with COVID-19, allowing comparison of screening approaches.

**Objective:**

To determine the prevalence of myocarditis in athletes with COVID-19 and compare screening strategies for safe return to play.

**Design, Setting, and Participants:**

Big Ten COVID-19 Cardiac Registry principal investigators were surveyed for aggregate observational data from March 1, 2020, through December 15, 2020, on athletes with COVID-19. For athletes with myocarditis, presence of cardiac symptoms and details of cardiac testing were recorded. Myocarditis was categorized as clinical or subclinical based on the presence of cardiac symptoms and CMR findings. Subclinical myocarditis classified as probable or possible myocarditis based on other testing abnormalities. Myocarditis prevalence across universities was determined. The utility of different screening strategies was evaluated.

**Exposures:**

SARS-CoV-2 by polymerase chain reaction testing.

**Main Outcome and Measure:**

Myocarditis via cardiovascular diagnostic testing.

**Results:**

Representing 13 universities, cardiovascular testing was performed in 1597 athletes (964 men [60.4%]). Thirty-seven (including 27 men) were diagnosed with COVID-19 myocarditis (overall 2.3%; range per program, 0%-7.6%); 9 had clinical myocarditis and 28 had subclinical myocarditis. If cardiac testing was based on cardiac symptoms alone, only 5 athletes would have been detected (detected prevalence, 0.31%). Cardiac magnetic resonance imaging for all athletes yielded a 7.4-fold increase in detection of myocarditis (clinical and subclinical). Follow-up CMR imaging performed in 27 (73.0%) demonstrated resolution of T2 elevation in all (100%) and late gadolinium enhancement in 11 (40.7%).

**Conclusions and Relevance:**

In this cohort study of 1597 US competitive athletes with CMR screening after COVID-19 infection, 37 athletes (2.3%) were diagnosed with clinical and subclinical myocarditis. Variability was observed in prevalence across universities, and testing protocols were closely tied to the detection of myocarditis. Variable ascertainment and unknown implications of CMR findings underscore the need for standardized timing and interpretation of cardiac testing. These unique CMR imaging data provide a more complete understanding of the prevalence of clinical and subclinical myocarditis in college athletes after COVID-19 infection. The role of CMR in routine screening for athletes safe return to play should be explored further.

## Key Points

**Question:**

What is the prevalence of myocarditis in competitive athletes after COVID-19 infection, and how would different approaches to screening affect detection?

**Findings:**

In this cohort study of 1597 US competitive collegiate athletes undergoing comprehensive cardiovascular testing, the prevalence of clinical myocarditis based on a symptom-based screening strategy was only 0.31%. Screening with cardiovascular magnetic resonance imaging increased the prevalence of clinical and subclinical myocarditis by a factor of 7.4 to 2.3%.

**Meaning:**

These cardiac magnetic resonance imaging findings provide important data on the prevalence of clinical and subclinical myocarditis in college athletes recovering from symptomatic and asymptomatic COVID-19 infections.

## Teaser

This cohort study assesses the prevalence of myocarditis in athletes with COVID-19 and compares screening strategies for safe return to play.

## Introduction

SARS-CoV-2, which causes COVID-19, has infected millions of people around the world, causing significant morbidity and mortality.^1^ Competitive athletes are a unique population that may be at high risk for environmental and situational transmission of disease, and once infected, may be at risk for sudden cardiac death (SCD) during training and competition.^2,3,4,5,6^ Viral myocarditis in asymptomatic people is a common cause of SCD, especially among those younger than 35 years.^7,8,9,10^ The incidence of SCD in collegiate athletes has been estimated at 1:50 000 per year. Even a small number of events in a young and apparently healthy population has devastating consequences. This often receives broad attention and, in some circumstances, may be preventable.^6,11^

Typical cardiac magnetic resonance (CMR) imaging findings of myocarditis or myocardial inflammation in asymptomatic or mildly symptomatic competitive athletes after COVID-19 infection have been described, even without other cardiac testing abnormalities.^12,13^ These reports describe variable estimates of myocarditis and myocardial inflammation prevalence (0%-15%).^12,14,15^ Such heterogeneity highlights the potential importance of CMR in detecting subclinical myocarditis (those without cardiac symptoms) and demonstrates the need for further investigation.^10,16,17,18^

Many schools and athletic conferences have developed screening protocols for safe return to play (RTP). Available consensus documents^19^ tie CMR and other cardiac testing to the presence of cardiac symptoms (symptoms-based screening strategy). Others require advanced testing for all athletes after COVID-19 infection. In September 2020, the Big Ten Conference mandated advanced testing for all athletes after COVID-19 infection prior to RTP, including electrocardiogram (ECG), echocardiogram, serum troponin level, and CMR imaging.^20^ Integral to this plan, the conference also formed the Big Ten COVID-19 Cardiac Registry for valid scientific data to inform RTP decisions.

The aims of this study were to estimate the prevalence of myocarditis among athletes after COVID-19 infection, to compare differences in COVID-19 myocarditis across Big Ten Universities, to evaluate the utility of different diagnostic strategies for myocarditis screening among competitive athletes, and to review timing and results of repeat CMR imaging to inform safe RTP decisions.

## Methods

The Big Ten COVID-19 Cardiac Registry is an observational study of athletes confirmed positive for SARS-CoV-2 by polymerase chain reaction testing. Thirteen of 14 Big Ten Universities agreed to participate (eAppendix 1 in Supplement 1). The present study was a survey (Supplement 2) of the experiences of the participating universities’ athletes with COVID-19 from March 1, 2020, through December 15, 2020, with focus on those who completed CMR imaging as part of their cardiac evaluation. Detailed, deidentified information from symptom questionnaires and advanced testing was reviewed for athletes with myocarditis. Data on age and race were not collected. The Ohio State University institutional review board serves as the central institutional review board for the Big Ten COVID-19 Cardiac Registry and approved this survey and waiver of consent. This report follows the Strengthening the Reporting of Observational Studies in Epidemiology (STROBE) reporting guideline.

### Survey Data

Each participating Big Ten University principal investigator reported the total number of athletes screened for SARS-CoV-2, the number with positive polymerase chain reaction results, the number completing cardiac screening with CMR imaging, and the number with findings that were consistent with myocarditis by the assessment of local clinicians. Restriction from training and competition by the local program was required for the diagnosis of myocarditis to be assigned. Those who did not complete CMR imaging as part of their cardiac evaluation or by data cut are described (eAppendix 2 in Supplement 1).

CMR findings consistent with myocarditis were classified based on updated 2018 Lake Louise criteria (LLC).^21^ A positive diagnosis was determined by presence of both T1-based criteria (T1 mapping, T1-weighted imaging, or late gadolinium enhancement [LGE]) and T2-based criteria (T2 mapping or T2-weighted imaging) in the same American Heart Association segment. LLC were modified by a requirement for colocalizing of Tl and T2 abnormalities to improve specificity and avoid interobserver variability. Although these criteria were agreed on in the Big Ten COVID-19 Cardiac Registry meeting, individual program clinicians determined the diagnosis of myocarditis. Diagnoses that deviated from these criteria are described in the Results section. Modified LLC also included supportive criteria such as pericardial effusion, pericardial inflammation, and left ventricular systolic dysfunction. Isolated right ventricular insertion point fibrosis was not used to diagnose myocarditis. For diagnoses consistent with myocarditis, details of the abnormal findings from CMR imaging, duration of days between COVID-19 diagnosis and CMR imaging, number and type of cardiac symptoms (chest pain, dyspnea on exertion [dyspnea], or chest palpitations), and number with abnormal findings on ECG or echocardiogram consistent with myocarditis or elevated troponin level (troponin was assessed according to assay and local laboratory standards as normal or elevated) were collected. Data collection for myocarditis diagnoses was limited to protect personal health information. Following survey submission, results were confirmed with the local principal investigator.

### Myocarditis Diagnosis Definitions

Myocarditis diagnoses were divided into 3 categories: (1) clinical myocarditis (cardiac symptoms present before or at the time of cardiac testing), (2) subclinical probable myocarditis (no cardiac symptoms) with abnormal ECG, echocardiogram, or troponin findings consistent with myocarditis, and (3) subclinical possible myocarditis (no cardiac symptoms) without abnormal ECG, echocardiogram, or troponin findings and only abnormal CMR imaging findings.

### Data Analysis

Data from Big Ten athletes who completed all recommended cardiac testing including CMR imaging were included in this analysis. Normality of distributions were tested using the Shapiro-Wilk test. Data are reported as counts (percentage) with qualitative descriptors and as mean (SD) or median (interquartile range) for continuous variables. Clinical/subclinical count data were analyzed using the Fisher exact test. There was variability in data collection methods among programs (eg, CMR scanners, acquisition protocols, readers, timing, etc) that may influence the detection of myocarditis and the frequency of myocarditis diagnoses across institutions. Therefore, in addition to the crude estimate of prevalence, an estimate of the percentage of athletes who received complete cardiac evaluation including CMR imaging affected by myocarditis was calculated using a generalized linear mixed-regression model (fixed effect for number of athletes with complete cardiac evaluation including CMR imaging and a random effect for institution) with a negative binomial distribution. 95% CIs were calculated using the Clopper-Pearson exact method for proportions for crude estimates and with standard methods for negative binomial distributions for the regression model. A sensitivity analysis was conducted including schools that performed CMR imaging in all cardiac evaluations before the Big Ten Conference mandate in September 2020 for CMR testing in all athletes after COVID-19 infections to assess possible selection bias on the overall observed prevalence of myocarditis. A *P* value less than .05 indicated statistical significance. All statistical analyses were performed using R version 4.0.3 (R Foundation for Statistical Computing).

## Results

**Figure 1.**
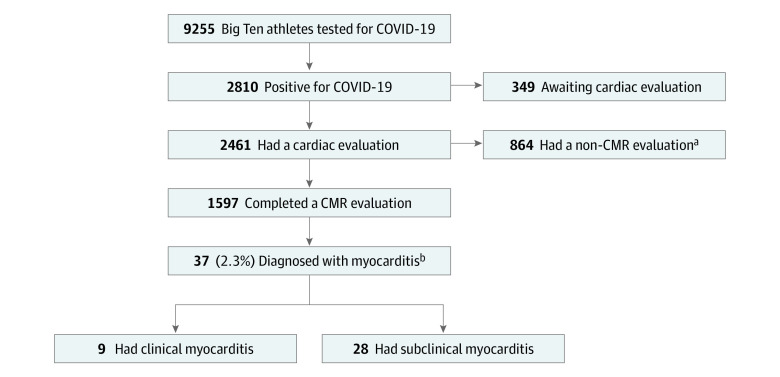
Cohort of Big Ten Athletes ^a^Athletes were excluded from analysis for not completing cardiac magnetic resonance (CMR) imaging as part of cardiac evaluation and described in more detail in eAppendix 2 in Supplement 1. ^b^Athletes diagnosed with myocarditis were categorized as clinical or subclinical based on presence or absence of cardiac symptoms.

Thirteen Big Ten Universities agreed to participate and submitted data. Through December 15, 2020, 9255 athletes had undergone COVID-19 testing and 2810 (30.4%) had tested positive. From this group of athletes with COVID-19 (1879 men [66.9%]), 2461 had completed cardiac evaluation, with 1597 (64.9%) including CMR imaging at the time of analysis and 864 (35.1%) with a non-CMR cardiac evaluation (eAppendix 2 in Supplement 1). Of those who had CMR imaging results, 37 athletes (2.3%) were diagnosed with either clinical or subclinical myocarditis (Figure 1).

### Myocarditis Diagnoses 

The 37 athletes were predominantly male (27) and represent 17 collegiate sports (8 women’s sports). Nine athletes with clinical myocarditis reported cardiac symptoms before or at the time of cardiac testing: 8 of 9 reported chest pain, 3 of 9 had dyspnea, and 3 of 9 had palpitations. There were 28 of 37 athletes with subclinical myocarditis who reported no cardiac symptoms. Of 28 athletes with subclinical myocarditis, 8 had abnormal cardiac testing other than CMR imaging and were classified as probable myocarditis: 1 of 28 had abnormal ECG findings, 3 of 28 had abnormal echocardiogram findings, and 4 of 28 had elevated troponin levels (Table). While 5 of 9 athletes with clinical myocarditis had abnormal additional testing results (ECG, echocardiogram, or troponin), only 8 of 28 with subclinical myocarditis had abnormal additional testing. Overall, 20 of 37 athletes had subclinical possible myocarditis who had no cardiac symptoms and nondiagnostic ECG findings, echocardiogram findings, and troponin level and therefore were only identified by meeting CMR imaging–modified LLC criteria or supportive criteria.

**Table.** Demographic, Imaging, and Biomarker Data for Athletes Diagnosed With Myocarditis^a^

Athlete	Cardiac symptoms	Troponin level	ECG findings	ECHO findings	Time from COVID-19 diagnosis, d	CMR imaging findings	Follow-up CMR imaging time and findings
**Clinical myocarditis**							
1	Chest pain, palpitations	Elevated	Abnormal	Abnormal	46	↑T2, LGE	12 wk; Residual LGE
2	Chest pain	Elevated	Abnormal	NCM	Unknown	↑T1, ↑T2, LGE	14 wk; Residual LGE
3	Chest pain, dyspnea	Normal	Abnormal	NCM	15	↑T2, LGE	10 wk; Resolved^b^
4	Chest pain, dyspnea	Normal	Abnormal	NCM	13	↑T2, LGE	12 wk; Residual LGE
5	Dyspnea	Normal	NCM	Abnormal	77	↓LVEF + pericarditis	Pending^c^
6	Chest pain, palpitations	Normal	NCM	NCM	25	LGE	Pending^c^
7	Chest pain	Normal	NCM	NCM	50	LGE	Pending^c^
8	Chest pain	Normal	NCM	NCM	25	↑T2, LGE	14 wk; Residual LGE
9	Chest pain, palpitations	Normal	NCM	NCM	45	↑T2, LGE	12 wk; Residual LGE
**Subclinical probable myocarditis**							
10	None	Elevated	NCM	NCM	30	↑T1,↑ T2, LGE	Pending^c^
11	None	Elevated	NCM	NCM	14	↑ T2, LGE	Pending^c^
12	None	Elevated	NCM	NCM	14	↑T2, LGE	12 wk; Residual LGE
13	None	Elevated	NCM	NCM	11	↑T2, LGE	4 wk; Residual LGE
14	None	Normal	Abnormal	NCM	13	↑T1,↑ T2, LGE	Pending^c^
15	None	Normal	NCM	Abnormal	42	↓LVEF, LGE	13 wk; Residual LGE
16	None	Normal	NCM	Abnormal	12	↓LVEF, LGE	4 wk; Resolved^b^
17	None	Normal	NCM	Abnormal	25	↑T1, ↑T2, LGE	Pending^c^
**Subclinical possible myocarditis**							
18	None	Normal	NCM	NCM	36	↑T2, LGE	13 wk; Residual LGE
19	None	Normal	NCM	NCM	20	↑T2, LGE	12 wk; Residual LGE
20	None	Normal	NCM	NCM	71	↑T2, LGE	10 wk; Resolved^b^
21	None	Normal	NCM	NCM	10	↑T2, LGE	10 wk; Residual LGE
22	None	Normal	NCM	NCM	14	↑T2, LGE	8 wk; Resolved^b^
23	None	Normal	NCM	NCM	11	↑T2, LGE	7 wk; Resolved^b^
24	None	Normal	NCM	NCM	11	↑T2, LGE	7 wk; Resolved^b^
25	None	Normal	NCM	NCM	15	↑T2, LGE	8 wk; Residual LGE
26	None	Normal	NCM	NCM	44	↑T2, LGE	6 wk; Residual LGE
27	None	Normal	NCM	NCM	21	↑T2, LGE	8 wk; Residual LGE
28	None	Normal	NCM	NCM	49	↑T2, LGE	10 wk; Resolved^b^
29	None	Normal	NCM	NCM	35	↑T2, LGE	6 wk; Resolved^b^
30	None	Normal	NCM	NCM	24	↑T2, LGE	6 wk; Residual LGE
31	None	Normal	NCM	NCM	51	LGE	4 wk; Resolved^b^
32	None	Normal	NCM	NCM	25	↑ T2, LGE	Pending^c^
33	None	Normal	NCM	NCM	20	↑T2, LGE	11 wk; Resolved^b^
34	None	Normal	NCM	NCM	48	↑T2, LGE	Pending^c^
35	None	Normal	NCM	NCM	14	↑T1, ↑T2, LGE	Pending^c^
36	None	Normal	NCM	NCM	11	↑T2, LGE	12 wk; Residual LGE
37	None	Normal	NCM	NCM	19	↑T2, LGE	10 wk; Resolved^b^

Abbreviations: CMR, cardiovascular magnetic resonance; ECG, electrocardiogram; ECHO, echocardiogram; LGE, late gadolinium enhancement; NCM, not consistent with myocarditis; ↓LVEF, decreased left ventricular ejection fraction; ↑T1, elevated T1 by T1 mapping or T1-weighted imaging based on individual institutional standards; ↑T2, elevated T2 by T2 mapping or T2-weighted imaging based on individual institutional standards.

^a^ A total 37 athletes (27 men), from 13 Big Ten Universities and across 17 sport disciplines were diagnosed with myocarditis. Of these 37 athletes, 9 athletes had cardiac symptoms (clinical myocarditis), and 28 athletes were asymptomatic (subclinical myocarditis). Further breakdown of the subclinical myocarditis group with those demonstrating abnormal cardiac testing outside of CMR imaging (subclinical probable myocarditis) and those with only CMR imaging abnormalities (subclinical possible myocarditis) is reported. Abnormal ECG and abnormal ECHO findings were defined by the program as consistent with myocarditis. Elevated troponin levels were defined by institutional standards and includes both troponin I and high-sensitivity troponin.

^b^ Both T1 and T2 abnormalities have resolved at follow-up CMR imaging.

^c^ Athlete is in recovery from COVID-19 myocarditis, and follow-up CMR imaging has not been performed.

### Diagnostic Approach

**Figure 2.**
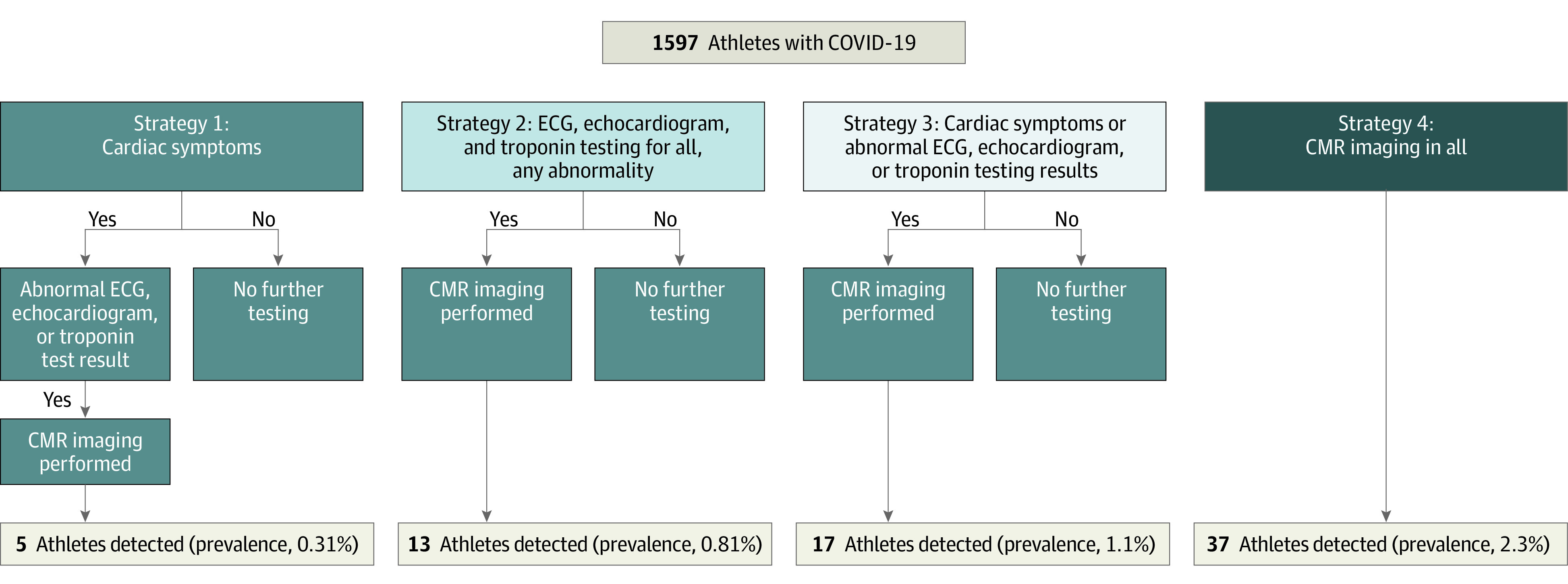
Detection and Estimated Prevalence of Myocarditis Based on Diagnostic Strategy From 37 athletes with clinical and subclinical myocarditis, the number that would have been detected and percentage prevalence found based on strategy performed and guided by either (1) cardiac symptoms alone; (2) electrocardiogram (ECG), echocardiogram, and troponin for all; (3) cardiac symptoms, ECG, echocardiogram, or troponin; or (4) cardiovascular magnetic resonance (CMR) imaging for all strategy.

Based on a published diagnostic strategy driven by cardiac symptoms,^19^ only 5 athletes (detected prevalence, 0.31%) of myocarditis would have been found in our cohort. A strategy using ECG, echocardiogram, and troponin findings regardless of cardiac symptoms with the addition of CMR imaging if any abnormality had been found, would have detected 13 athletes (detected prevalence, 0.81%). A strategy using CMR imaging in all athletes after COVID-19 infection regardless of cardiac symptoms or other cardiac testing results increased the prevalence to 2.3%, a 7.4-fold increase from the symptom-driven strategy and 2.8-fold increase over the ECG, echocardiogram, and troponin strategy (Figure 2).

### CMR Findings

**Figure 3.**
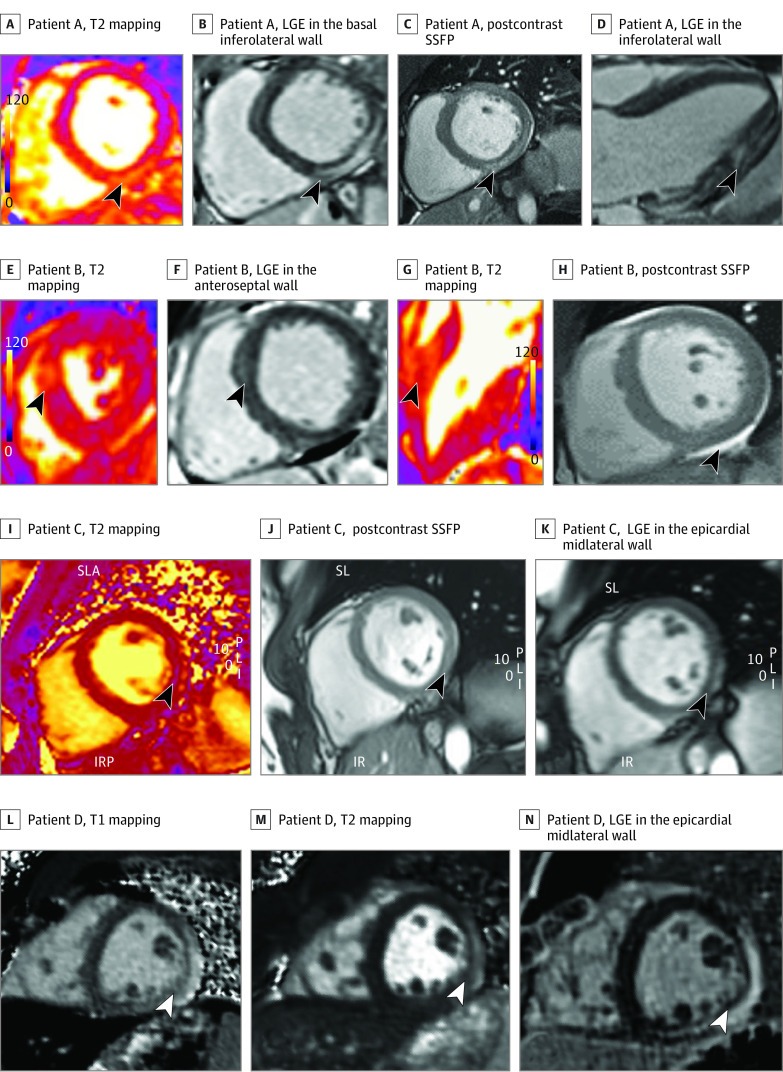
Cardiac Magnetic Resonance Imaging in Athletes With Clinical and Subclinical Myocarditis A-D, Athlete A with subclinical possible myocarditis was asymptomatic with normal electrocardiogram (ECG), echocardiogram, and high-sensitivity troponin findings. A, T2 mapping showing elevated T2 in basal-mid inferolateral wall in short axis view. B, late gadolinium enhancement (LGE) in the basal inferolateral wall in short axis view. C, Postcontrast steady state-free precession (SSFP) images showing contrast uptake in the basal-mid inferolateral wall in short axis view. D, LGE in the inferolateral wall in 3-chamber view. E-H, Athlete B with subclinical probable myocarditis was asymptomatic with normal ECG, normal echocardiogram, and elevated high-sensitivity troponin findings. E, T2 mapping showing elevated T2 in the anteroseptal wall in short axis view. F, LGE in the anteroseptal wall in 3-chamber view. G, T2 mapping showing elevated T2 in the anteroseptal wall in 3-chamber view. F, Postcontrast SSFP image showing pericardial effusion in short axis view. I-K, Athlete C with clinical myocarditis and chest pain, dyspnea, abnormal ECG, normal echocardiogram, and normal troponin findings. I, T2 mapping showing elevated T2 in the lateral wall short axis view. J, Postcontrast SSFP images showing contrast uptake in midlateral wall in short axis view. K, LGE in the epicardial midlateral wall in short axis view. L-N, Athlete D with clinical myocarditis, chest pain, abnormal ECG, echocardiogram, and troponin findings. L, T1 mapping showing elevated native T1 in midlateral wall in short axis view. M, T2 mapping showing elevated T2 in the midlateral wall in short axis view. N, LGE in the epicardial midlateral wall in short axis view. IR indicates inferior right view; IRP, inferior, right, posterior view; PLI, posterior, left, inferior view; SL, superior left view; SLA, superior, left, anterior view.

Overall, 31 of 37 CMR imaging findings reported as myocarditis met the modified LLC^21^ with elevated T2 and elevated T1 or LGE in the same location (American Heart Association myocardial segment). Of the 6 athletes not meeting modified LLC criteria, 3 had clinical myocarditis. One athlete reported dyspnea; echocardiogram and CMR imaging demonstrated decreased left ventricular systolic function (left ventricular ejection fraction, 35%-40%); in addition, CMR imaging showed pericardial inflammation and effusion. The 2 other athletes with clinical myocarditis reported chest pain and had CMR imaging with LGE in a pattern typical for myocarditis. Of the 3 athletes with subclinical myocarditis and CMR imaging not meeting modified LLC, 2 demonstrated significantly reduced left ventricular systolic function and LGE in patterns typical of myocarditis, thus meeting supportive LLC. In the third athlete, CMR imaging demonstrated extensive LGE in a typical pattern for myocarditis without myocardial edema on T2 mapping. Modified LLC and CMR imaging findings were not significantly different between subclinical (25 of 28 [89.3%]) vs clinical (6 of 9 [66.7%]) (difference = 22.6% [95% CI, −7.1% to 57.7%; *P* = .14) (Figure 3).

In follow-up, 27 of 37 athletes (73.0%) completed repeat CMR imaging with a range of 4 to 14 weeks (mean [SD], 9.4 [3.1] weeks) from initial COVID-19 test positivity. Two patterns emerged at CMR imaging follow-up. The first was complete resolution of both T2 mapping abnormalities and LGE in 11 of 27 athletes (40.7%; range between studies, 4-10 weeks with median [interquartile range] of 8 [3.5] weeks). The second was resolution of T2 mapping abnormalities but persistence of LGE in 16 of 27 athletes (59.3%; range between studies, 4-14 weeks with median [interquartile range] of 12 [4.3] weeks) (eFigure in Supplement 1).

One of 6 athletes with clinical myocarditis showed complete CMR imaging resolution (both T2 elevation and LGE resolved), with the second CMR study performed 10 weeks after diagnosis. In comparison, 10 of 21 athletes with subclinical athletes with myocarditis (47.6%) demonstrated complete resolution of inflammation and LGE with a mean (SD) time after diagnosis of 7.7 (2.5) weeks (range, 4-11 weeks).

Additional abnormalities identified on CMR imaging included 46 athletes with LGE alone (either focal and not meeting American Heart Association segment criteria, or right ventricular insertion point LGE) without elevated T1 or T2, 34 athletes with pericardial abnormalities, and 4 athletes of pulmonary infiltrates. Detailed analysis of pattern of LGE in athletes without myocarditis was not performed.

### Big Ten Universities and Variability

**Figure 4.**
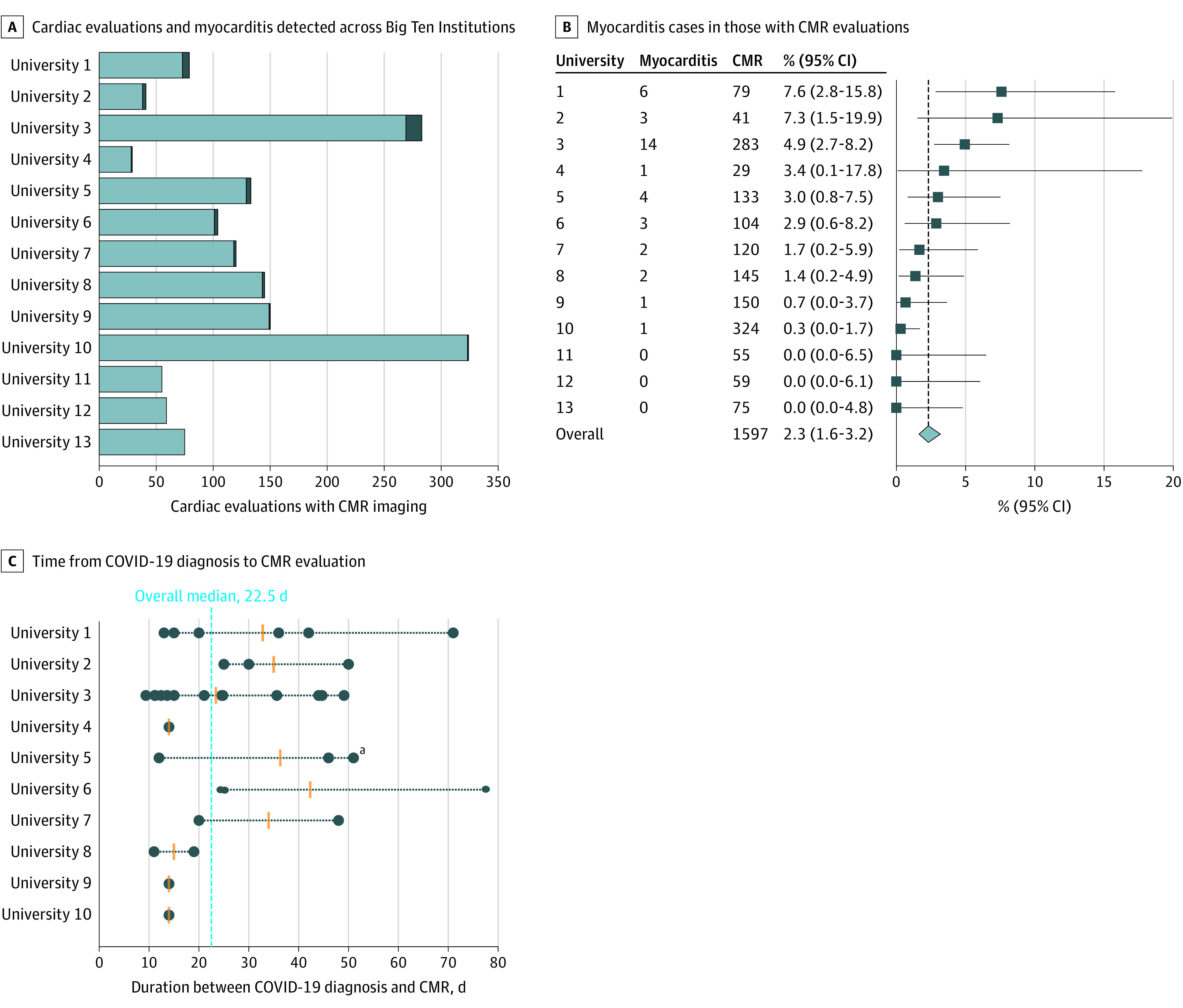
Cardiac Evaluations Performed in Big Ten Athletes Cardiovascular magnetic resonance (CMR) imaging, athletes diagnosed with myocarditis, and CMR timing after COVID-19 diagnosis in Big Ten athletes with recent SARS-CoV-2 infection. A, The reported number of normal (light blue) and athletes diagnosed with myocarditis (dark blue) observed from complete cardiac evaluations including CMR imaging completed in Big Ten athletes with recent SARS-CoV-2 infection. B, The reported number of athlethes diagnosed with myocarditis, complete cardiac evaluations including CMR imaging, and the percent myocarditis with the 95% CI (calculated using the Clopper-Pearson exact method) for each participating university and the overall prevalence (crude estimate, calculated as the quotient of all athletes with myocarditis and CMR imaging performed across all universities). C, Duration between COVID-19 diagnosis and CMR imaging for athletes who were diagnosed with myocarditis (n = 36). Data are displayed for participating institution where cases of myocarditis were observed (n = 10). ^a^Duration data unknown for 1 athlete diagnosed with myocarditis. Filled circles represent individual case duration, and orange horizontal lines represent the institution median duration between COVID-19 diagnosis and CMR imaging.

Reported findings varied among Big Ten Universities, including (1) COVID-19 positivity rate (overall, 30.4%; range, 13.0%-48.2%), (2) timing of complete cardiac testing, and (3) prevalence of myocarditis. Figure 4A shows the number of athletes with COVID-19 per program who had complete cardiac testing results available including CMR imaging (median [interquartile range], 104 [86]; range, 29-324) and those with myocarditis. The prevalence of myocarditis per program ranged from 0% to 7.6% (overall, 2.3% [95% CI, 1.6%-3.2%]; model-based estimate, 2.1% [95% CI 1.1%-4.4%]), with 3 institutions reporting 0 cases and 10 programs reporting at least 1 case of myocarditis (Figure 4B). In the subgroup analysis including schools where CMR imaging was performed in all cardiac evaluations (n = 5), from 919 cardiac evaluations, 21 athletes were diagnosed with myocarditis, resulting in a prevalence of 2.3% (95% CI, 1.4-3.5%), consistent with the overall observed prevalence.

The timing from COVID-19 test positivity to cardiac testing and diagnosis of myocarditis ranged between 10 and 77 days (median [interquartile range], 22.5 [28.5] days) (Figure 4C).

## Discussion

In this study of aggregate data from 13 Big Ten Universities, 1597 athletes who tested positive by polymerase chain reaction for SARS-CoV-2 underwent comprehensive cardiac evaluation including ECG, echocardiogram, troponin, and CMR imaging. Of these, 2.3% had either clinical or subclinical myocarditis that restricted them from training and competitive play. Allowing for potential differences in CMR performance and distributional characteristics yielded an estimated prevalence of myocarditis of 2.1% (95% CI, 1.1%-4.4%).

Myocarditis is a significant risk factor for SCD in athletes, especially at younger ages.^7,9,18^ In an autopsy study of US Air Force recruits with SCD, physical activity was a risk factor, and the most common suspected underlying factor was unrecognized myocarditis.^18^ In another study, lymphocytic myocarditis was a common cause of ventricular arrhythmias.^10^ Several studies have shown that physical exertion leads to worsening disease and eventual death in mouse models of myocarditis.^22,23,24^

Myocardial injury during SARS-CoV-2 infection is well described in clinically distinct populations.^25,26,27^ Early assessments in athletes with COVID-19 demonstrated myocardial injury and inflammation in this otherwise healthy cohort, prompted the Big Ten Conference to integrate advanced cardiac screening into RTP protocols to reduce risk to student athletes.^12,28^ The Big Ten COVID-19 Cardiac Registry was formed to analyze available clinical data to identify and reduce risk to student athletes, to inform RTP decisions, and to further scientific understanding on the cardiac effect of SARS-CoV-2.^20^

Our prevalence estimates differ from those of several other reports.^12,13,14,15,29^ In addition to random error, several sources of ascertainment bias may contribute to the observed variability. Timing of CMR imaging after COVID-19 infection (eFigure in Supplement 1), variability in CMR imaging hardware and software, technique, protocol, and expertise in interpretation may all distort estimates across programs using comparable screening protocols (eAppendix 3 in Supplement 1). Differences in screening approaches may also yield much different estimates of the prevalence of myocarditis. Our data demonstrate the increased detection of clinical and subclinical myocarditis when CMR imaging is added to previously published screening protocols. (Figure 2).

Although details regarding circumstances and in particular symptoms prior to the incident event of SCD are not known in most cases, some athletes may be asymptomatic or minimally symptomatic at the time of the event. In a recent study reviewing SCD in athletes due to autopsy-proven myocarditis, more than 50% had no reported symptoms (viral and/or cardiac), and only 16 of 74 (21.6%) reported cardiac symptoms (chest pain, palpitations, syncope) prior to the event.^30^ Additionally, if risk to the athlete is related to myocardial abnormalities (inflammation, edema, fibrosis), our preliminary evaluation demonstrated similar CMR findings (T2 mapping and LGE) between baseline clinical vs subclinical myocarditis and with or without abnormalities on cardiac testing (Figure 3). Beyond the acute issues and concerns, there may be benefit to establishing baseline myocardial abnormalities after COVID-19 to determine the need for follow-up studies and future risk. However, this inference would require more detailed expert CMR imaging core analysis and more cases to determine if there are myocardial inflammatory patterns and differences in LGE that would be clinically useful.

CMR abnormalities resolve for some cases, which seems clinically encouraging. Although the number of cases are too few for analysis with adequate statistical power, clinical myocarditis cases in this sample appear less likely to demonstrate resolution of LGE than subclinical cases, at least within the period of follow-up reported here. While the lack of resolution in those cases might have been expected, the limited follow-up data we present also suggests that longer intervals between initial and follow-up CMR imaging do not always result in resolution of CMR findings.

Use of CMR screening for all athletes who have had SARS-CoV-2 infection is challenging for many reasons. Data are lacking on the prevalence of CMR changes that could be related to athletic cardiac adaptation.^31,32^ Moreover, the LLC used in CMR diagnosis of myocarditis have been validated only in symptomatic myocarditis cases and not systematically studied in an asymptomatic cohort.^21,32,33,34^ Therefore, using CMR imaging as a screening tool to detect subclinical myocarditis after a viral infection warrants further analysis and continues to be a work in progress. This will require short- and long-term outcome data to clarify the implications of these findings.

Although CMR imaging was completed as part of cardiac evaluation for fewer than 100% of the Big Ten athletes with COVID-19, selection bias associated with our estimate of myocarditis prevalence appears unlikely for 3 reasons. First, some athletes did not undergo CMR imaging because prior to the mandate, it was not required. Hence, the reason for missing CMR imaging data was independent of relation to symptoms or other testing and may reasonably be treated as uninformatively censored. Second, our sensitivity analysis conducted among the 5 programs that completed CMR imaging in all athletes resulted in a prevalence of 2.3% (95% CI, 1.4%-3.5%) is consistent with the overall observed myocarditis prevalence for all programs (2.3%; 95% CI, 1.6%-3.2%). Third, the other 8 programs added complete cardiac evaluations and CMR imaging for all athletes at various times, all after the mandate. All clinical and subclinical probable myocarditis cases from these 8 programs were diagnosed after the mandate, when the programs had incorporated CMR imaging as part of the cardiac evaluation.

The data presented suggest that 1.8% of athletes with prior SARS-CoV-2 infection are both asymptomatic and have subclinical myocarditis. Importantly, inclusion of CMR imaging in the RTP protocol also has the advantage of reassuring that myocardial injury has not occurred if the CMR findings are normal. CMR imaging is highly sensitive for identifying myocardial inflammation^21,34,35^ and in our study was able to exclude significant disease and allow safe RTP in 97.7% of athletes after cardiac screening. While there may be a concern that CMR imaging is too sensitive and therefore unduly restrict athletes from sport, such a scenario would only account for a very small proportion of the population based on our study.

In our view, the role of CMR imaging in routine screening for athletes’ safe RTP should be explored further; we could then better assess the possible risk to those athletes with undiagnosed subclinical myocarditis who exercise and the benefit of ruling out significant myocardial inflammation and injury by a normal CMR.

### Limitations

Not all athletes who tested positive for SARS-CoV-2 infection underwent CMR imaging evaluation prior to September 2020, when the Big Ten Conference mandated comprehensive cardiac testing for all athletes. It is possible that prior to this period, there could have been a selection bias in undergoing CMR imaging. However, our review of this population (eAppendix 2 in Supplement 1) and sensitivity analysis suggests that selection bias in referral to CMR imaging was not likely to influence the observed prevalence of myocarditis in this study. Sources of CMR variability as described may have influenced prevalence estimates and require standardization. LLC were developed in a different clinical population, so generalizability of these criteria to this context of SARS-CoV-2–infected population may be flawed and requires further analysis. There are several other concerns regarding using CMR imaging as a screening tool. CMR imaging may not be easily accessible to all, the volume may exceed local capacity, local expert interpretation may be insufficient, and CMR imaging could be considered costly.

Detailed, person-specific biographical data were not collected. Our survey and observational study are intended to be an institution-level analysis with a nested case series of athletes who had CMR abnormalities consistent with myocarditis to allow us to report prevalence of myocarditis. Individual-level information and COVID-19–negative athletes will be an important addition to future CMR imaging comparative analysis. Uniform and validated evaluation of diagnostic data in core laboratories has not occurred at this time. Thus, local evaluations of diagnostic data may be inconsistent with validated interpretations.

## Conclusions

Among Big Ten athletes with recent SARS-CoV-2 infection and complete cardiac screening prior to RTP, 2.3% had evidence of clinical or subclinical myocarditis. We observed variability in prevalence across universities, and this may be based on timing of CMR imaging relative to COVID-19 infection and variability in CMR protocols and interpretation. In our study, testing protocols are closely tied to the detection of myocarditis, and cases may not have been detected without CMR imaging. Further detailed core analysis will guide CMR screening protocols and Big Ten RTP recommendations. At present, we do not know the natural history or the short- and long-term implications to an athlete with COVID-19 clinical or subclinical myocarditis. To address these concerns, we must find ways to minimize the variability in performance among academic centers to diagnose myocarditis, perhaps through standardized evidence-based diagnostic algorithms and testing protocols, and when indicated, standardization of CMR protocols and interpretation. These unique CMR data give us a more complete understanding of the prevalence of clinical and subclinical myocarditis in college athletes recovering from symptomatic and asymptomatic COVID-19 infections. The Big Ten COVID-19 Cardiac Registry is committed to longitudinal study and elucidating the best role of CMR imaging in returning athletes to sport after COVID-19 infection.

## Notes

Supplement 1. eAppendix 1. The Big Ten Conference and Participating Universities eAppendix 2. Non-CMR Cardiac Evaluation Cohort eAppendix 3. Big Ten Cardiac Magnetic Resonance Imaging Protocols eFigure. Follow up CMR Findings for Athletes Diagnosed with Myocarditis (n=27)

Supplement 2. Big Ten Cardiac Registry Survey

Supplement 3. **Nonauthor Collaborators. **Big Ten COVID-19 Cardiac Registry Investigators.
