# Supplementary material for: Prevalence of Clinical and Subclinical Myocarditis in Competitive Athletes With Recent SARS-CoV-2 Infection: Results From the Big Ten COVID-19 Cardiac Registry
Source: JAMA Cardiol. 2021 May 27;6(9):1078–87. doi: 10.1001/jamacardio.2021.2065 (PMC8160916; doi:10.1001/jamacardio.2021.2065)
Supplement: Supplement 1. — eAppendix 1. The Big Ten Conference and Participating Universities eAppendix 2. Non-CMR Cardiac Evaluation Cohort eAppendix 3. Big Ten Cardiac Magnetic Resonance Imaging Protocols eFigure. Follow up CMR Findings for Athletes Diagnosed with Myocarditis (n=27) [file jamacardiol-e212065-s001.pdf]

Supplemental Online Content

Daniels CJ, Rajpal S, Greenshields JT, et al; Big Ten COVID-19 Cardiac Registry Investigators. Prevalence of clinical and subclinical myocarditis in competitive athletes with recent SARS-CoV-2 infection: results from the Big Ten COVID-19 Cardiac Registry. *JAMA Cardiol*. Published online May 27, 2021. doi:10.1001/jamacardio.2021.2065

eAppendix 1. The Big Ten Conference and Participating Universities
eAppendix 2. Non-CMR Cardiac Evaluation Cohort
eAppendix 3. Big Ten Cardiac Magnetic Resonance Imaging Protocols
eFigure. Follow up CMR Findings for Athletes Diagnosed with Myocarditis (n=27)

This supplemental material has been provided by the authors to give readers additional information about their work.

© 2021 Daniels CJ et al. *JAMA Cardiology.*

eAppendix 1. The Big Ten Conference and Participating Universities

The Big Ten Athletic Conference is comprised of 14 preeminent institutions that share a common goal of pursuing academic and athletic excellence. It has more than 9,600 student- athletes and sponsors 28 sport disciplines for men and women. Thirteen universities participated in this study.

Indiana University

Michigan State University

Northwestern University

Ohio State University

Penn State University

Purdue University

Rutgers University

University of Iowa

University of Maryland

University of Michigan

University of Minnesota

University of Nebraska

University of Wisconsin

© 2021 Daniels CJ et al. *JAMA Cardiology.*

eAppendix 2. Non-CMR Cardiac Evaluation Cohort

From Figure 1, 2810 athletes were COVID positive, 2461 had completed cardiac testing at the time of the survey and data cut. From the 2461 COVID-19 positive athletes, 1597 had completed cardiac evaluation that included CMR and 864 had a non-CMR cardiac evaluation. The focus of the survey and observational study was to evaluate athletes that completed CMR as part of their cardiac evaluation and determine the prevalence of CMR abnormalities consistent with myocarditis. As such, we did not collect detailed data on the 864 non-CMR cardiac evaluations. There are several sources for this population and all evaluations were determined at institutional level. 1) Athletes evaluated before the Big Ten mandated CMR in all COVID-19 positive athletes. 2) Athletes several weeks or months from COVID-19 infection. 3) Pandemic restrictions for athletes to travel crossing state lines for CMR. 4) Dedication of local resources for performing CMR to on-campus actively training and competing athletes. 5) Backlog of athletes not completing CMR at the time of the survey. Based upon several factors including our sensitivity analysis comparing the 5 programs that completed CMR in all athletes resulting in a prevalence of 2.3%, 95% CI 1.4 to 3.5%, is consistent with the overall observed prevalence for all programs (2.3%, 95% CI 1.6-3.2 %), and that in the other 8 programs, all clinical and subclinical probable myocarditis cases were diagnosed after the mandate, when programs had incorporated CMR as part of the cardiac evaluation, minimizes any effect from these cases. But, the lack of detailed information is a limitation.

© 2021 Daniels CJ et al. *JAMA Cardiology.*

eAppendix 3. Big Ten Cardiac Magnetic Resonance Imaging Protocols

We performed a survey of Big Ten programs cardiac magnetic resonance (CMR) protocols. The majority of programs performed CMR studies at a field strength of 1.5 T (12/13). Three sites also imaged at 3.0T, and one program exclusively performed studies at 3.0T. CMR experience ranged from 2 programs performing 5-10 CMRs per week, 5 programs performing 10-20 CMRs per week and 6 programs reported performing >20 CMRs weekly. While 12 programs included performance of T1 and T2 mapping for athlete scans, 1 institution reported using only T1 weighted imaging. One institution did not have availability of mapping sequences for their scanner. Values for native T1, T2 and ECV at 1.5T were 1030-1100 msec, 50-60 msec, and 29-32%, respectively based on local institutional cutoffs. Values for 3.0T were 1250-1300 msec, 42-55 msec, and 30-32%, respectively.

All programs performed Cine SSFP and LGE while only 7 reported performing first pass perfusion. While most programs reported performing basal and mid short axis T1 and T2 mapping or T2 weighted imaging, long axis T1 and T2 mapping were performed by only 50% of the CMR labs. Similarly, while short axis LGE stacks (either single shot or multi-breath hold) was performed consistently by all institutions, there was variability in performance of long axis LGE. Only 6 programs completed detailed mapping and LGE.

© 2021 Daniels CJ et al. *JAMA Cardiology.*

eFigure. Follow up CMR Findings for Athletes Diagnosed with Myocarditis (n=27)

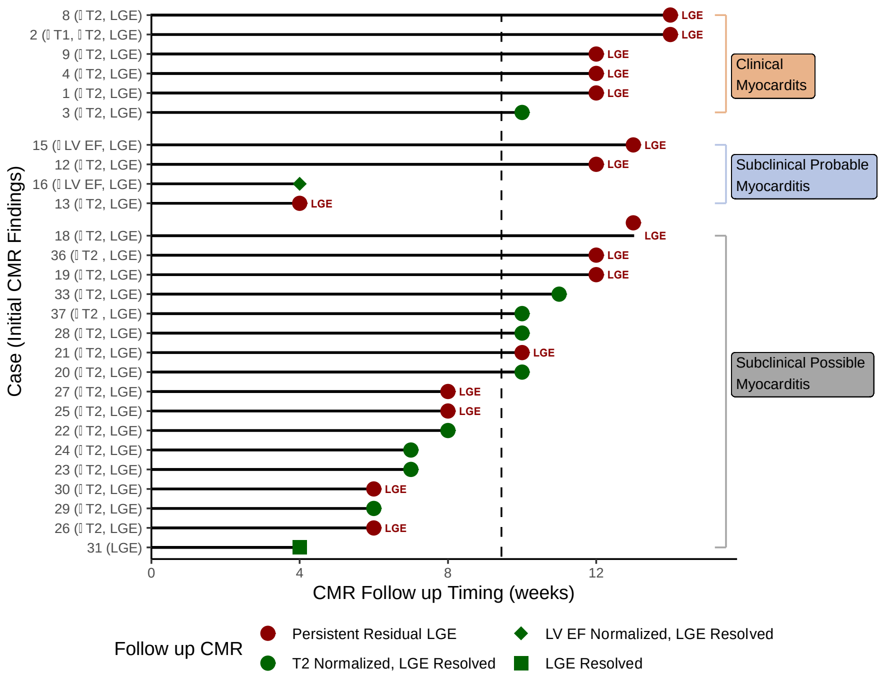

eFigure 1 Legend: The horizontal axis displays the duration between the initial CMR and follow up CMR, the vertical axis displays case number and the initial CMR findings in parentheses. Cases are categorized into three groups: Clinical Myocarditis (n = 6), Subclinical Probable Myocarditis with abnormal ECG, ECHO, or troponin in addition to CMR abnormalities (n = 4), and Subclinical Possible Myocarditis with CMR abnormalities only (n = 17). Follow up CMR showed resolution in 11 cases (T2 Normalized, LGE Resolved, n = 9; LV EF Normalized, LGE Resolved, n = 1; LGE Resolved, n = 1), persistent residual LGE was observed in 16 cases. The overall average follow-up duration (9.4 weeks) is represented by the vertical dashed line. Abbreviations: ↑T2 = Elevated T2 by T2 mapping or T2 weighted imaging based on individual institutional standards, ↑T1 = Elevated T1 by T1 mapping or T1 weighted imaging based on individual institutional standards, LGE = Late gadolinium enhancement on CMR, LVEF= left ventricular ejection fraction

© 2021 Daniels CJ et al. *JAMA Cardiology.*
