# Supplementary material for: Prevalence of Clinical and Subclinical Myocarditis in Competitive Athletes With Recent SARS-CoV-2 Infection: Results From the Big Ten COVID-19 Cardiac Registry
Source: JAMA Cardiol. 2021 May 27;6(9):1078–87. doi: 10.1001/jamacardio.2021.2065 (PMC8160916; doi:10.1001/jamacardio.2021.2065)
Supplement: Supplement 3. — Nonauthor Collaborators. Big Ten COVID-19 Cardiac Registry Investigators. [file jamacardiol-e212065-s003.pdf]

Supplemental Online Content: Nonauthor Collaborators

*Indicates required information. Only first name, last name, and suffix will appear in PubMed.

*Group Name(s): Big Ten COVID-19 Cardiac Registry							
***First Name and Middle Initial(s)**	***Last Name**	***Suffix (eg, Jr, III)**	Academic Degrees	Institution	Location (city, state/province, country)	Role or Contribution, eg, chair, principal investigator	Group (if more than 1 Group listed in the byline) and/or Subgroup (eg, Steering Committee)
Orlando	Simonetti		PhD	The Ohio State University			
Karolina	Zareba		MD	The Ohio State University			
Salman	Bhatti		MD	The Ohio State University			
Daniel	Addison		MD	The Ohio State University			
Timothy	Obarski		MD	The Ohio State University			
Emile	Daoud		MD	The Ohio State University			
Matthew	Granger		APN	The Ohio State University			
Suzanne	Smart		BS	The Ohio State University			
Jessica	Mayercin-Johnson		MA	The Ohio State University			
Preethi	Subramanian		MS	The Ohio State University			
Jeffery	Glitt		MSW	The Ohio State University			
Deborah	Mitchell		RN	The Ohio State University			
Rose	Chumita		RN	The Ohio State University			
Amy	Mumford		RN	The Ohio State University			
Anne	Garcia		BS	The Ohio State University			
Lori	Garris		RN	The Ohio State University			
Hongjie	Liu		PhD	University of Maryland			
Bradley	Hatfield		PhD	University of Maryland			
Yuji	Zhang		PhD	University of Maryland			
Douglas	Boersma		MS	Pudue University			
Zachary	Schlader		PhD	Indiana University			
Shawn	Goodwin		MS	Indiana University			
Nicholas	Port		PhD	Indiana University			
Taylor	Zuidema		MS	Indiana University			
Jennifer	Maldonado		BS	University of Iowa			
Lee	Eckhardt		MD	University of Wisconsin			
Scott	Reeder		MD	University of Wisconsin			
Mathue	Baker		MD	Bryan Heart (University of Nebraska)			

Supplemental Online Content: Nonauthor Collaborators

*Indicates required information. Only first name, last name, and suffix will appear in PubMed.

***First Name and Middle Initial(s)**	***Last Name**	***Suffix (eg, Jr, III)**	Academic Degrees	Institution	Location (city, state/province, country)	Role or Contribution, eg, chair, principal investigator	Group (if more than 1 Group listed in the byline) and/or Subgroup (eg, Steering Committee)
Wayne	Sebastianelli		MD	Penn State University			
Rebecca	Wadlinger		MD	Penn State University			
Roberta	Millard		MD	Penn State University			
Philip	Bosha		MD	Penn State University			
Haley	Sunday			Penn State Health			
Danae	Steele			Penn State Health			
Anisa	Chaudhry		MD	Penn State Health			
Soraya	Smith		MD	Penn State Health			
Micheal	Pfeiffer		MD	Penn State Health			
John	Kellerman		MD	Penn State Health			
Gregory	Billy		MD	Penn State University			
Jason	Krystofiak		MD	Rutgers University			
Micah	Eimer		MD	Northwestern Medicine			
